# High-Velocity Impact Performance of Ballistic Fabric Using Core-Spun Compound Yarns

**DOI:** 10.3390/polym16212973

**Published:** 2024-10-23

**Authors:** Dan Yang, Shengdong Liu, Weitian Zhang, Qian Liu, Gaozheng Yao, Kai Zhu

**Affiliations:** 1 School of Materials Science and Engineering, Changzhou University, Changzhou 213164, China; 15108900177@163.com (S.L.); 18325573383@163.com (W.Z.); beichen680501@163.com (Q.L.);; 2 National Experimental Demonstration Center for Materials Science and Engineering, Changzhou University, Changzhou 213164, China

**Keywords:** Kevlar^®^ filament yarns, core-spun compound yarns, ballistic performance, FEA model

## Abstract

In this paper, the usage of core-spun compound yarns in ballistic fabric to improve ballistic performance is considered, as with the use of core-spun compound yarns, the yarn friction inside the fabric is enhanced, and, therefore, the energy absorption capability of the fabric is expected to increase. Three types of fabric were developed and compared. F_a_ refers to a woven type made with 100% Kevlar^®^ filament yarns. F_b_ was woven with core-spun compound aramid yarns, which were made of Kevlar^®^ filament yarns spun with staple aramid fiber. F_c_ was woven with core-spun compound polyester yarns, which were made of Kevlar^®^ filament yarns spun with staple polyester fiber. There were two main purposes for comparing these types. The first was to confirm if the ballistic performance could be improved with the usage of core-spun compound yarns instead of pure filament yarns. The second was to investigate if different compositions of spun fiber would influence ballistic performance. The research results are positive and quite interesting. They show that the usage of core-spun compound yarn could indeed help to increase ballistic performance and that core-spun compound aramid yarns are better than core-spun compound polyester yarns in this function. The research was carried out using both ballistic tests and FEA models.

## 1. Introduction

Conflicts and wars have always existed in the long history of the human world. Inadequate safety measures could place our frontline professionals at unnecessary risk and cause intolerable injury or harm. Various types of materials have been used to protect humans from injuries in battles and other dangerous situations throughout history [1]. Ancient tribes used animal skins and other natural materials to protect their bodies. The warriors of ancient Rome and medieval Europe covered their torsos in metal plates before entering battle. However, with the advent of more effective weapons, e.g., guns and cannons, most traditional materials seem unreliable or too heavy to be effective against firearms [2,3,4,5,6,7,8].

Currently, with the development of scientific research, high-performance materials, such as aramid yarn (the famous brand known as Kevlar^®^) and UHMWPE yarn (the famous brand known as Dyneema^®^), have been developed [9,10,11,12]. Fabrics made of these materials are important ballistic materials because of their high tenacity, high modulus, light weight, and other super advantages. Furthermore, structure plays a fundamental role in determining the ballistic performance of the whole ballistic fabric. As a highly recognized type, ballistic material with a woven structure has been widely researched [13,14,15,16,17].

When a projectile impacts woven fabric, two types of yarns are often distinguished and observed. One type is primary yarns, which are directly hit by the projectile and play a major role in absorbing ballistic energy. The other is secondary yarns, which are not directly hit by the projectile but also influenced because of their interaction with the primary yarns due to the friction function. When these yarns in the fabric are pressed against a projectile, the impact energy could be converted into various forms, including yarn failure, fiber pull-out, fiber fracture, cone formation on the back face of the target, and so on [17,18,19].

Impact energy conversion is governed by several factors, such as the material properties of the constituent fibers, the boundary conditions of the sample fabric, the projectile’s geometry, and so on. Among all these, the factor of friction is quite important. An increased interyarn friction may require more impact energy to overcome, which would, therefore, lead to an increase in the ability to absorb impact energy [20]. As a matter of fact, many researchers have investigated the influence of friction on ballistic performance [21,22,23,24].

Chu and Rahman found that higher interyarn friction in woven fabric could help with areas involved in ballistic energy absorption via both experimental and numerical methods [25,26]. Zhou and Sun developed a yarn-gripping method to increase the interyarn friction, which could improve the resulting fabric’s capabilities of ballistic energy absorption and dissipation [27,28]. Briscoe and Motamedi discovered that if the interyarn friction was high in woven fabric, the fabric’s absorption and dissipation of ballistic energy could be more effective [29,30]. Yogita et al. fabricated aligned Ag nanordos (AgNRs) on a Kevlar fabric surface to increase the interyarn friction, thereby improving ballistic performance [31]. Mukesh et al. used shear thickening fluid (STF) to increase the inter-yarn friction and thereby enlarge the bearing area of the fabric; the fabric’s ballistic energy absorption was correspondingly enhanced [32,33,34,35,36].

## 2. Fabrication

**Figure 1:**
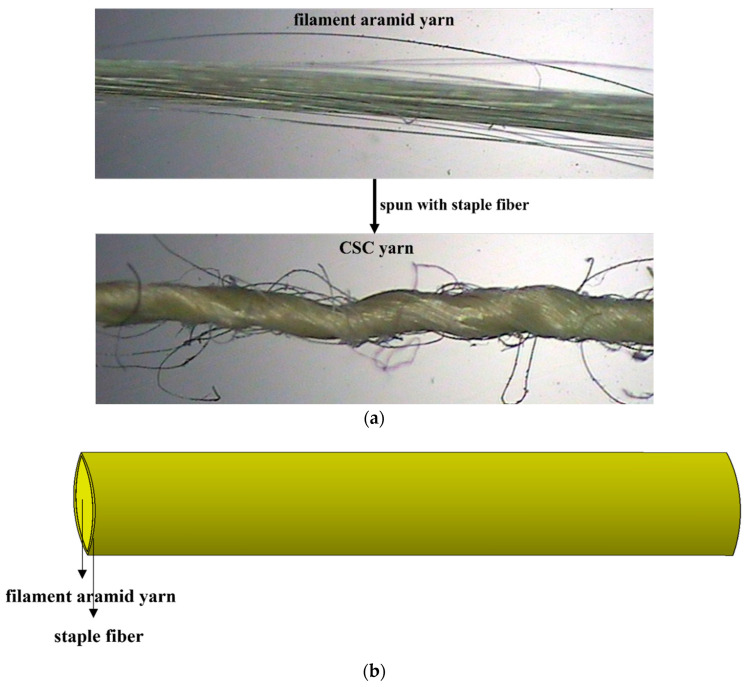
CSC yarn: (**a**) observation under SEM, (**b**) illustration.

**Figure 2:**
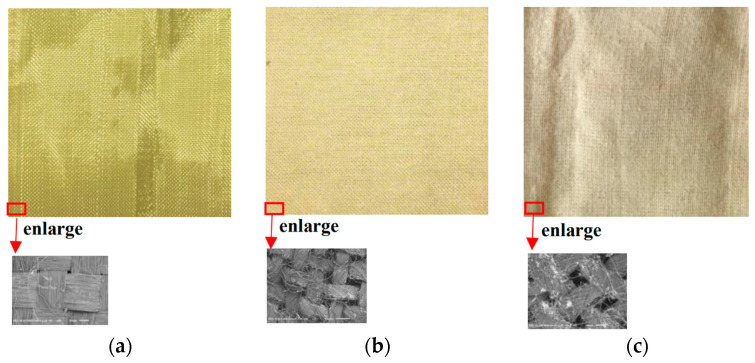
Manufactured fabrics: (**a**) F_a_, (**b**) F_b_, (**c**) F_c_.

In this paper, core-spun compound (CSC) yarn was considered to enhance the friction, and therefore to increase the ballistic performance, of the whole fabric. As shown in Figure 1, this CSC yarn was made of filament aramid yarn as the core yarn and spun with coarse staple fiber. Better interyarn friction was expected in a structure composed of CSC yarns than in one composed of conventional smooth filament aramid yarns only. Additionally, different compositions of staple fiber were considered to observe if this factor would influence ballistic performance. Three types of woven fabrics were manufactured. F_a_ is a conventional woven fabric using Kevlar^®^ 29 filament yarns. F_b_ was woven with CSC yarns, the coarse staple fiber of which was aramid. F_c_ was also woven with CSC yarns, but the coarse staple fiber was polyester. The manufactured F_a_, F_b_, and F_c_ are shown in Figure 2. F_a_ was very shiny, as it was made of 100% Kevlar^®^ 29 filament yarn; F_b_ was not so shiny, as aramid staple fiber was used; and F_c_ was inclined to be white, as the polyester staple fiber was white. The specification of these fabrics is listed in Table 1.

## 3. Ballistic Test

**Figure 3:**
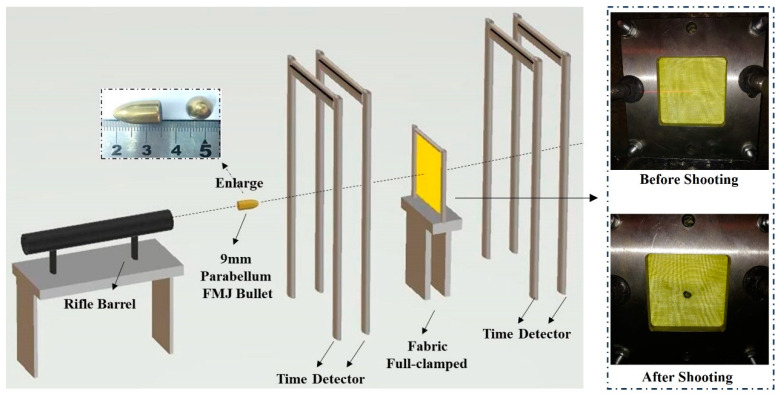
Schematic diagram of testing apparatus.

The samples were ballistically tested at the impact velocity ranging from 230 m/s to 330 m/s in the ballistic laboratory. The ballistic apparatus consisted of a firing device with a 7.62 mm rifle barrel, the target holder and time detectors in an enclosed environment, as shown in Figure 3. Propelled by gunpowder, the rifle barrel fired the projectile, which penetrated the full-clamped fabric fixed on the target holder. The projectile was a 9 mm Parabellum FMJ bullet with a weight of 8 g. The time detectors picked up the travelling time of the projectile before and after it penetrated the fabric to calculate the impact velocity and the exit velocity. The loss in kinetic energy carried by the projectile after penetrating the fabric was calculated using the following equation: Δ*E* = 1/2 (*mV*_0_^2^) − 1/2 (*mV*_1_^2^), where Δ*E* is the loss of kinetic energy, *m* is the projectile mass, *V*_0_ is the impact velocity, and *V*_1_ is the exit velocity.

**Figure 4:**
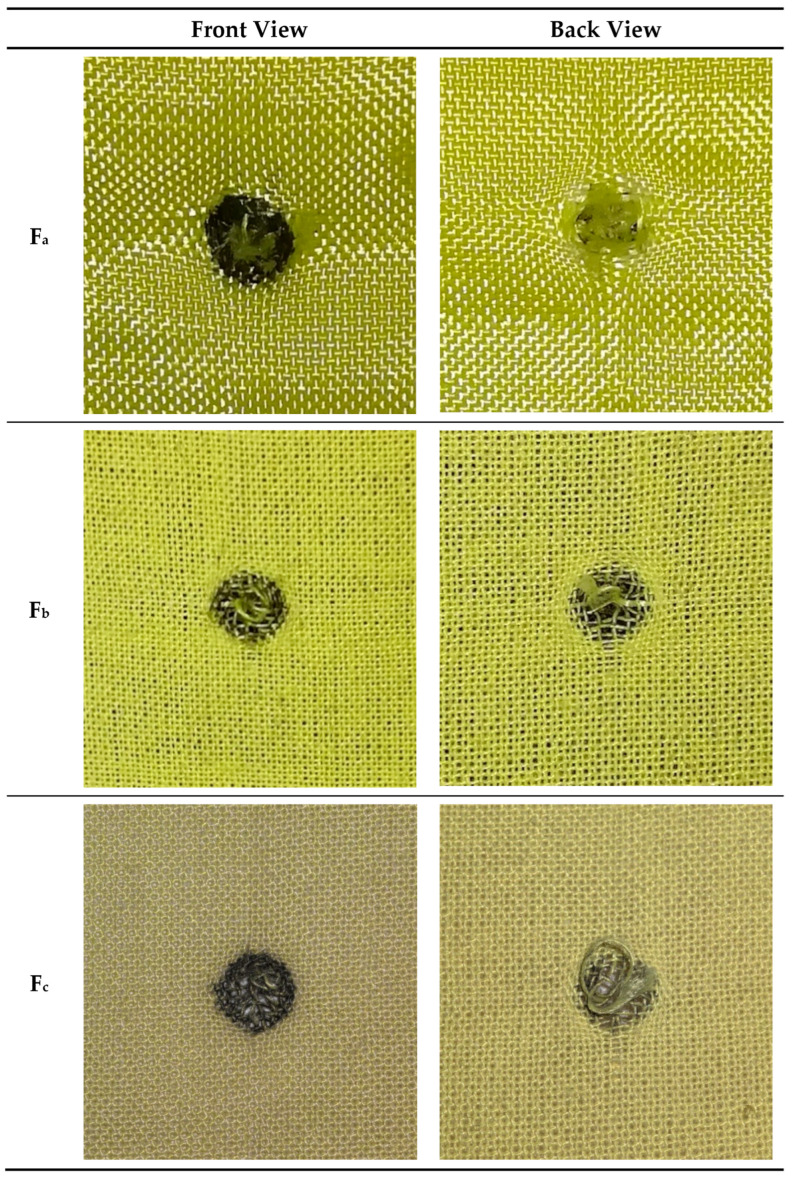
Appearance after fabrics ballistic tested.

The ballistic appearance of the impacted fabrics is shown in Figure 4. The universal ballistic phenomenon such as yarn failure, yarn pulled-out, and yarn fracture also could be discovered on the fabrics of F_a_, F_b_, and F_c_. A similar range of velocities from 220 m/s to 320 m/s was chosen to shoot F_a_, F_b_, and F_c_ to compare their ballistic performance in the same test conditions. In order to reduce the error of comparisons, their ballistic test results need to be normalized by the areal density.

**Figure 5:**
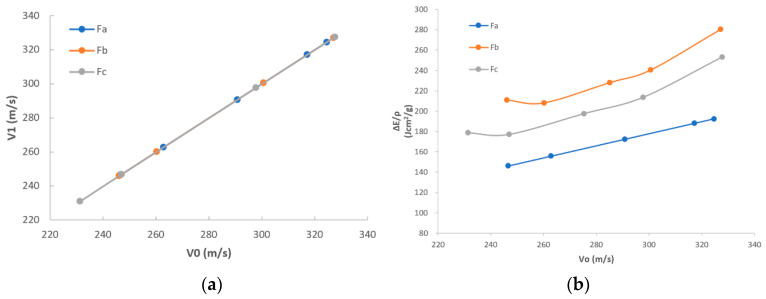
Comparisons among F_a_, F_b_, and F_c_: (**a**) V_0_ vs. V_1_; (**b**) V_0_ vs. energy after normalization.

In Figure 5a, it can be seen that the overall trend of impact velocity (marked as $E_{1}$) vs. exit velocity (marked as ΔE) is similar among F_a_, F_b_, and F_c_. This may indicate that with the increase in $E_{1}$, F_a_, F_b_, and F_c_ show similar variations of ΔE. Additionally, ΔE increases following the tendency of $E_{1}$, as the similar stable trend with no fluctuation is discovered for F_a_, F_b_, and F_c_. It could be concluded that there is no certain special value that could apparently influence the results, as the difference in velocity before and after the fabric is shot is inclined to be unchanged.

In Figure 5b, the first eye-catching point is that F_b_ and F_c_ are better than F_a_, and F_b_ is the highest, which indicates that F_b_ has the best energy absorption, and F_c_ ranks second while F_a_ is the lowest, regardless of whether under the low impact velocity or the high impact velocity. This may support the concept stated in the beginning of this paper that the usage of the core-spun compound (CSC) yarn could enhance the friction and therefore the ballistic performance of the whole fabric. The key factor is that, compared to 100% filament yarns, CSC yarns are apparently much coarser and more friction is expected. F_a_ is interlaced by Kevlar^®^ 29 filament yarns, which are shiny and smooth. F_b_ is interlaced by CSC aramid yarns which are made by Kevlar^®^ filament yarns spun with staple aramid fiber. F_c_ is interlaced by CSC polyester yarns which are made by Kevlar^®^ filament yarns spun with staple polyester fiber. Regardless of whether staple aramid fiber or staple polyester fiber, they can increase the friction among the yarns inside the woven structure, especially in the interlacements or yarn-to-yarn conjunctions, and, therefore, they help improve the energy absorption. It is known that the frictional work dissipated at the yarn-to-yarn junctions is a critical factor in determining the static tensile yarn and transverse fabric stiffness [29,30]. Frictional processes within the fabric systems are important for both normal indentation and ballistic deformations as they control the effective stiffness of the material. The fabrics with high friction dissipated the larger amounts of energy relative to fabrics with lower friction.

Additionally, the different composition of staple fibers also can have a different influence on the energy absorption capability, as F_b_ is better than F_c_. This may because the aramid staple fiber used in F_b_ has larger strength than the polyester staple fiber used in F_c_. This may indicate that apart from the factor of friction between the staple fiber and the core filament yarn, the strength of staple fiber also affects the ballistic performance of the whole fabric.

In Figure 5b, it can be further seen that there is a general trend for all F_a_, F_b_, and F_c_; more kinetic energy can be observed if the impact velocity is increased. This is because regardless whether it is F_a_, F_b_, or F_c_, the main component composing the yarn is the Kevlar^®^ 29 filament fiber. It has characteristics of high strength and high modulus, and has better capability of kinetic energy absorption under high-impact speed than that under low-impact speed.

This may be a good research conclusion to prove that the introduction of the core-spun compound (CSC) yarn is an ideal solution to increase the friction and, therefore, to provide better ballistic performance to the whole fabric.

## 4. FEA Model

Apart from the ballistic test, the ballistic performances of F_a_, F_b_, and F_c_ were further theoretically analyzed and compared in detail by establishing the full-size microstructure FEA models with the assistance of finite element software LS-DYNA R11.

### 4.1. Establishment of Geometrical Model with Mesh Scheme

It is well known that the basic component to establishing an FEA model is the geometrical model. In this project, the model of the projectile and the model of fabric are the two main parts of the geometrical model. The model of the projectile is built based on the parameters of 9 mm Parabellum FMJ bullet, provided by the Institute 53 of China Ordnance Industry Group in Jinan city of China. This is a kind of standard bullet verified by the international ballistic NIJ standard. The model of fabric is built based on assembling the models of warp and weft yarns. Because both the bullet and the fabric are axisymmetric structures, a 1/4 geometrical model is used in this project as it is enough to reflect the evaluated ballistic event as well as improve calculation efficiency.

**Figure 6:**
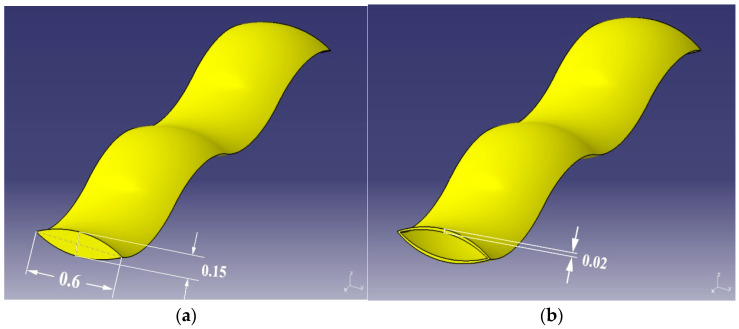
Basic model: (**a**) single-yarn, (**b**) tube.

The basic single-yarn model is illustrated as a three-dimensional solid object with an oval cross-section with certain crimp wave for both the warp yarn and the weft yarn, as shown in Figure 6. The major axis and the minor axis for the oval cross-section of single-yarn model are set as 0.6 mm and 0.15 mm, according to the observation. This basic single-yarn model is further wrapped by the tube with the thickness around 0.02 mm, representing the part of staple fiber spun tightly and wholly on the CSC yarn, which is further composed to form F_b_ and F_c_.

The geometrical models were further meshed using Hypermesh software 2020. Hypermesh is one of the most popular softwares dealing with finite elements codes, because of its computational efficiency and strong robustness. There were 30,720 elements for the F_a_ model, 61,440 elements for the F_b_ model, and the same for the F_c_ model, as F_b_ and F_c_ are only different in the composition of staple fiber spun on the CSC yarn. The geometrical model of projectile was established based on the parameters of a 9 mm Parabellum FMJ bullet, which was then meshed with 435 hexahedron elements on the body and 199 tetrahedron elements at the bottom.

**Figure 7:**
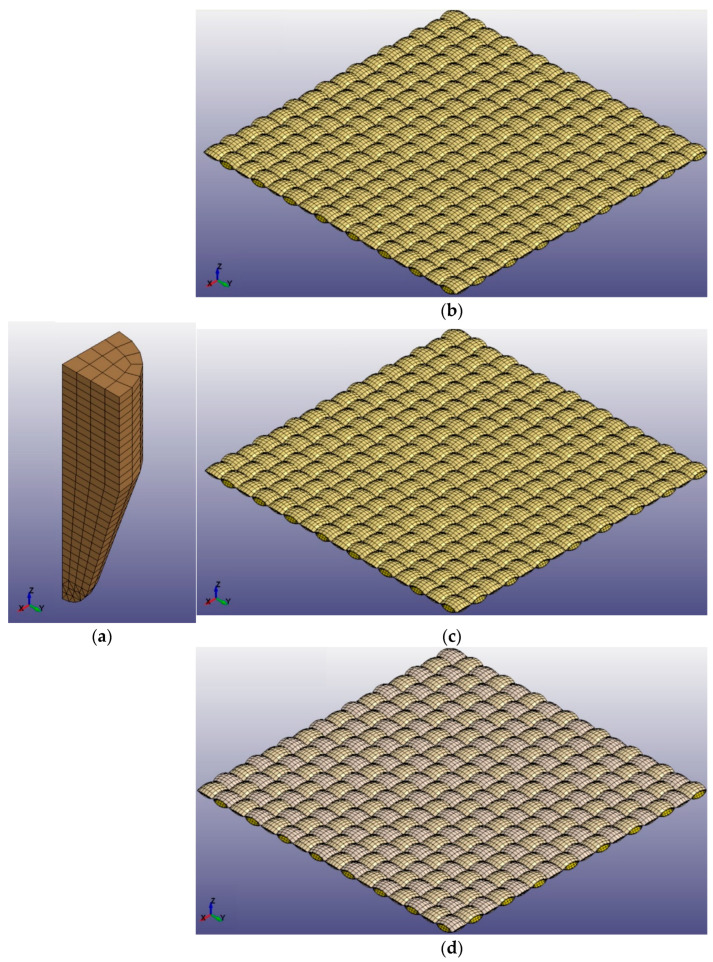
1/4 FEA models: (**a**) bullet, (**b**) F_a_, (**c**) F_b_, (**d**) F_c_.

The meshed geometrical model was further processed to complete the establishment of the FEA model by setting definition of material properties, definition of section properties, and application of boundary conditions and velocity to the bullet. All the information was saved as K files in LS-DYNA software. The 1/4 FEA models for F_a_, F_b_, and F_c_ with a bullet are individually shown in Figure 7.

### 4.2. Verification of FEA Model

#### 4.2.1. Observation of Phenomena

**Figure 8:**
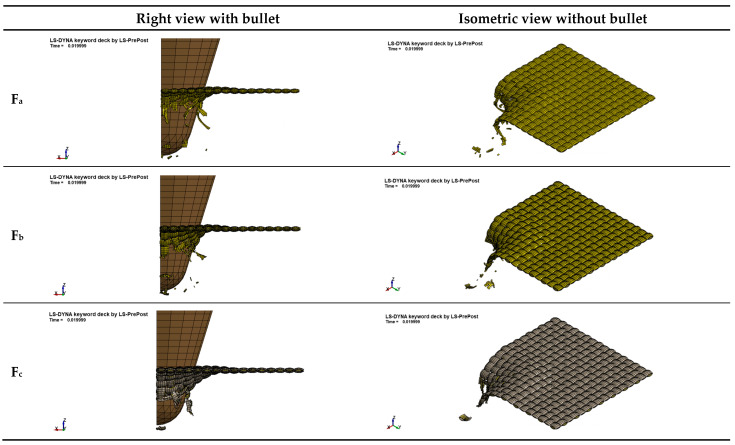
Illustration by FEA models.

Figure 8 shows that fabrics under ballistic test were successfully illustrated by the FEA model. It could be discovered that yarn failure, yarn fracture, and yarn pull-out, which are major characteristics found in experimental testing, could also be clearly shown by the FEA model. This is because of the activities of two typical waves. According to the energy absorption mechanism, when the projectile hits the fabric, two types of waves (longitudinal wave and transverse wave) come out and propagate through primary yarns and secondary yarns. The longitudinal wave travels through primary yarns, which are directly hit by the projectile, and secondary yarns, which are not directly hit by the projectile but interact with primary yarns in the plane of the target plate. The transverse wave propagates outwards from the impact zone to make deformation in the perpendicular direction to the fabric plane, leading to the phenomena including yarn failure, yarn fracture, and yarn pull-out that could be observed at the location hit by the projectile.

#### 4.2.2. Data Analysis

**Figure 9:**
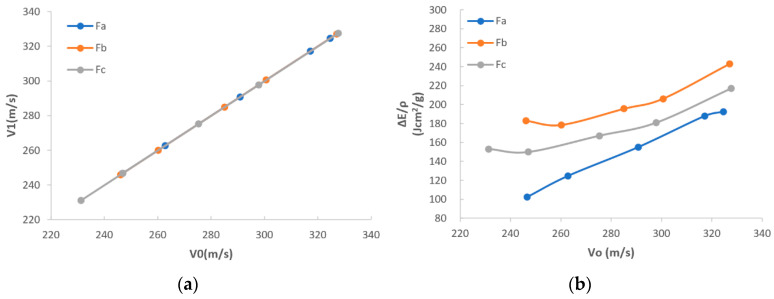
Comparisons among samples F_a_, F_b_, and F_c_ with theoretical data: (**a**) V_0_ vs. V_1_; (**b**) V_0_ vs. energy after normalization.

Excepted for the phenomena, a good agreement between the ballistic test result (shown in Table 2) and the FEA model result (shown in Table 3) was found. The trends of theoretical data are shown in Figure 9, which are also similar with the trends of experimental data shown in Figure 5.

Figure 9a illustrates the comparison between impact velocity (marked as $E_{1}$) and exit velocity (marked as ΔE). The same $E_{1}$ is applied for both the ballistic test and the FEA model, and very close trends are found when ΔE is varied, which verifies the validation of the FEA model. Additionally, it was discovered that ΔE increases stably with the increase in $E_{1}$. This may indicate that the variation of velocity before and after fabric penetrated tends to be stable and not influenced by a certain special value.

Figure 9b illustrates the comparisons between $E_{1}$ and $V_{1}$, known as impact velocity and loss of kinetic energy, respectively, calculated by FEA models for three types of fabrics F_a_, F_b_, and F_c_. The first eye-catching point is that the difference of trends between the ballistic test results, shown in Figure 5b, and the FEA model results, shown in Figure 9b, is quite tiny, which demonstrates, again, the validity of the FEA model. The reason for this tiny difference is that the real yarn has thousands of fibers but is simplified as a continuum surface with thickness in the FEA model. The non-simultaneous failure of each filament and complex interface friction may lead to a complicated energy absorption mechanism in the real experiment. Figure 9b also shows that F_b_ and F_c_ have better capability of energy absorption than F_a_, and F_b_ is the best regardless of whether at low impact velocity or high impact velocity. This may theoretically prove that with the introduction of short fibers, the ballistic performance of the fabric could be apparently improved due to the enhancement of the friction. Apart from that, in Figure 9b, F_b_ is shown to be better than F_c_, which also theoretically proves that the difference in composition of staple fiber can have an influence on the ballistic performance of the fabric. Compared to F_a_, the superiorities of F_b_ and F_c_ are further theoretically analyzed from the aspects of energy absorption, penetration resistance, and stress distribution, which are mainly important to demonstrate the ballistic performance of fabrics.

### 4.3. Theoretical Discussion

#### 4.3.1. Energy Absorption

The value of energy absorption is an important index to estimate the ballistic performance of a certain fabric and, further, to demonstrate the influence of structure difference on the ballistic performance. The energy carried by the bullet is transformed to the fabric through the form of yarn pull-out, yarn failure, or yarn fracture with the propagation of longitudinal wave and transverse wave when the projectile hits the fabric.

**Figure 10:**
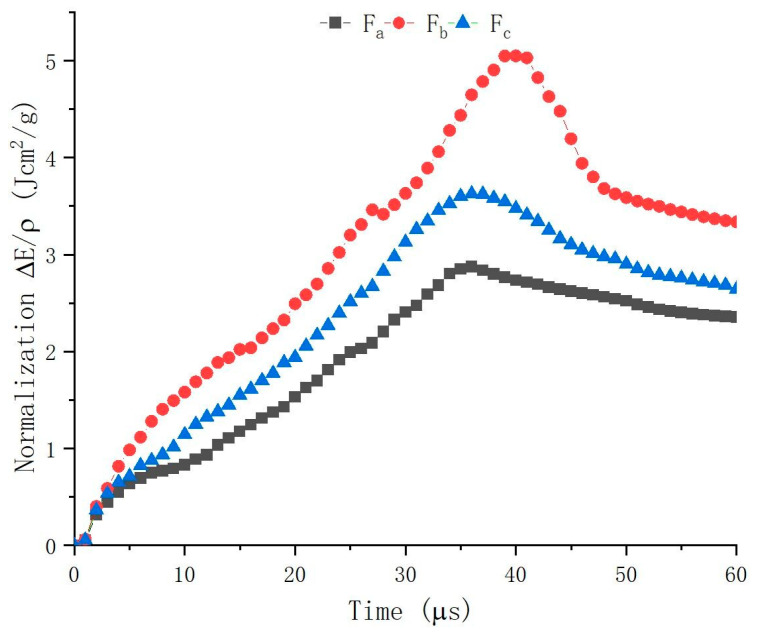
Comparisons of energy absorption in total.

The energy absorption of the fabric can be calculated according to the following equation: Δ*E* = 1/2 (*mV*_0_^2^) − 1/2 (*mV*_1_^2^) and is mainly reflected in the energy absorbed by the warp and weft yarns. The samples of F_a_ with impact velocity 290.8 m/s, F_b_ with impact velocity 300.6 m/s, and F_c_ with impact velocity 297.8 m/s were chosen for comparison as they have similar impact velocities, but F_c_ and F_b_ have larger energy absorption than F_a_, and F_b_ shows the highest, which demonstrates the overall trend, as shown in Figure 9. The comparisons among them may be more convincing. The main discoveries are found as follows:

The energy absorption of yarns in total for F_a_, F_b_, and F_c_ are compared to analyze if the introduction of CSC yarns could increase the ballistic performance during different times, as shown in Figure 10. The first apparent point is that regardless of which time, F_b_ and F_c_ are always higher than F_a_ apparently. The energy absorption in total shown in Figure 9b also demonstrates the same result. This may theoretically emphasize that F_b_ and F_c_ are superior to F_a_ in terms of ballistic performance with the usage of CSC yarns.

Secondly, F_b_ is better than F_c_, as shown in Figure 10. This may indicate that aramid staple fiber is better than polyester staple fiber to form CSC yarns in the construction of ballistic woven fabric, as the former can not only provide friction among yarns in the structure, but can also give better strength than the latter at the same time.

Apart from that, for F_a_, F_b_, and F_c_, the peak values of energy absorption in total are different, as shown in Figure 10. For F_a_, the trend increases steeply, arrives at the peak value around 35 µs, and then decreases gradually. However, the whole procedure of F_a_ is still comparatively smoother, compared to F_b_ and F_c_. The peak value of F_a_ is apparently lower than that of F_b_ and F_c_. This may indicate that during the whole procedure against the projectile penetration, the capability of energy absorption of F_a_ is always weaker than F_b_ and F_c_ and cannot surpass them at any moment. For F_b_ and F_c_, both increase steeply in the first period of 30 µs, then F_c_ stays at its peak value around 3.5 Jcm^2^/g around 35 µs and decreases gradually, while F_b_ fluctuates apparently and achieves its peak value around 5 Jcm^2^/g at 40 µs. The difference in the composition of staple fiber to form CSC yarns may be the reason for the difference between F_b_ and F_c_.

**Figure 11:**
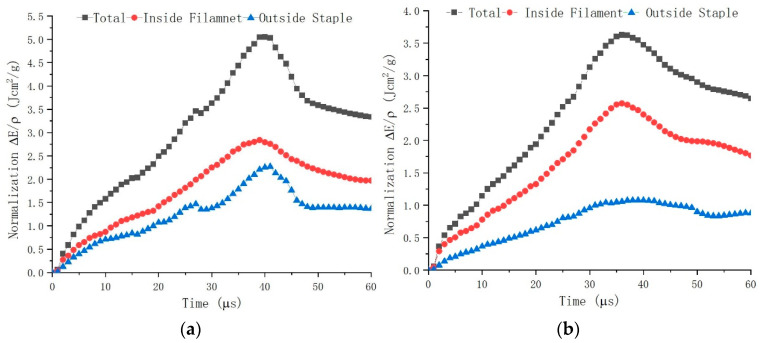
Analysis of energy absorption in different parts: (**a**) F_b_, (**b**) F_c_.

As a matter of fact, the ballistic performance of the fabric is, indeed, influenced by the different outside staple fibers used in the CSC yarns though the same Kevlar^®^ 29 filament yarn used inside. The composition of the outside staple fiber is aramid and polyester for F_b_ and F_c_, respectively, and the corresponding energy absorption capability is varied differently. As can be seen in Figure 11a, the value of aramid staple fiber increases smoothly until 25 µs, then increases steeply at 40 µs and finally decreases, which demonstrates a similar trend as the total trend. In Figure 11b, the value of polyester staple fiber increases very stably until 35 µs and then decreases slowly, which is totally different from the trend of the value of aramid staple fiber shown in Figure 11a. In addition, it can be noticed that regardless of the staple fiber used, either aramid or polyester, the value of filament shows a similar trend in both Figure 11a,b; it increases slowly until at the top value around 2.5 Jcm^2^/g, and then decreases slowly. This may indicate that the difference in outside staple fiber may have little influence on the energy absorption capability of the inside filament.

#### 4.3.2. Penetration Resistance

**Figure 12:**
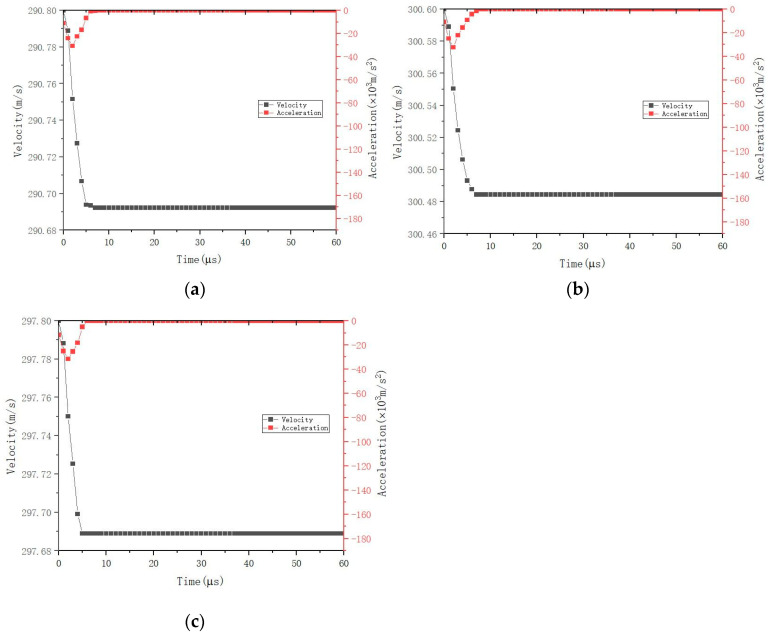
Velocity and acceleration vs. time history: (**a**) F_a_, (**b**) F_b_, (**c**) F_c_.

Apart from the energy absorption, the penetration resistance is another significant index to demonstrate the ballistic performance of the fabric. In this project, the situation of penetration resistance of F_a_, F_b_, and F_c_ is shown in Figure 12. It can be noticed that F_a_, F_b_, and F_c_ decrease sharply from the start to around 5–7 μs, and then remain unchanged. This may indicate that the projectile penetrates all types of fabrics quite successfully and completely. They show the similar trends which indicate that the same basic structure, namely, woven structure, is the fundamental factor to influence their ballistic performance though different yarns used. The woven structure is interlaced by warp and weft yarns. Both weft and warp yarns resist this penetration in the formation of stretching, deformation, pull-out, and fracture. After 7 μs, all whole structures have been completely penetrated. After that, very tiny and even no penetration resistance appears, and the velocity also tends to be unchanged and stable. The only difference is that F_b_ needs a little more time to achieve its lowest velocity (around 7 μs), compared to F_a_ and F_c_ (around 5 μs). This is because the staple fiber used for F_b_ is aramid staple, which can provide better friction than the polyester fiber used for F_c_ and no staple fiber for F_a_ to increase the interaction of ballistic mechanism in the structure.

**Figure 13:**
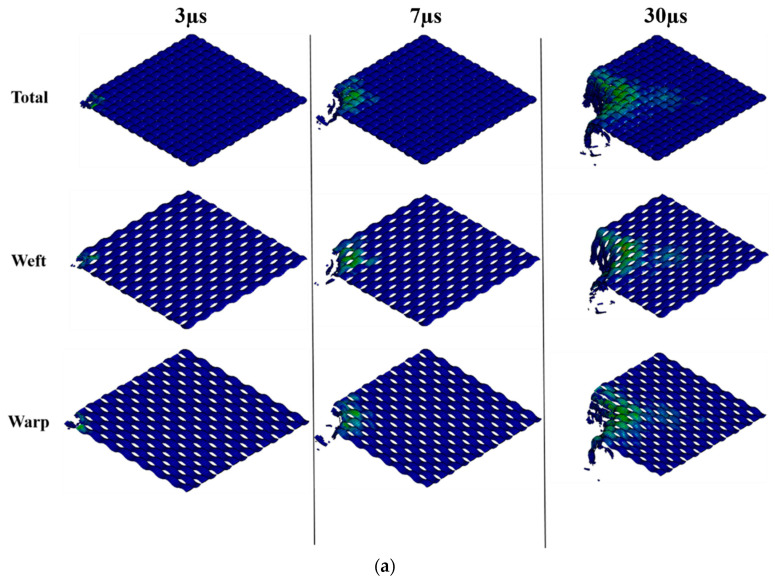

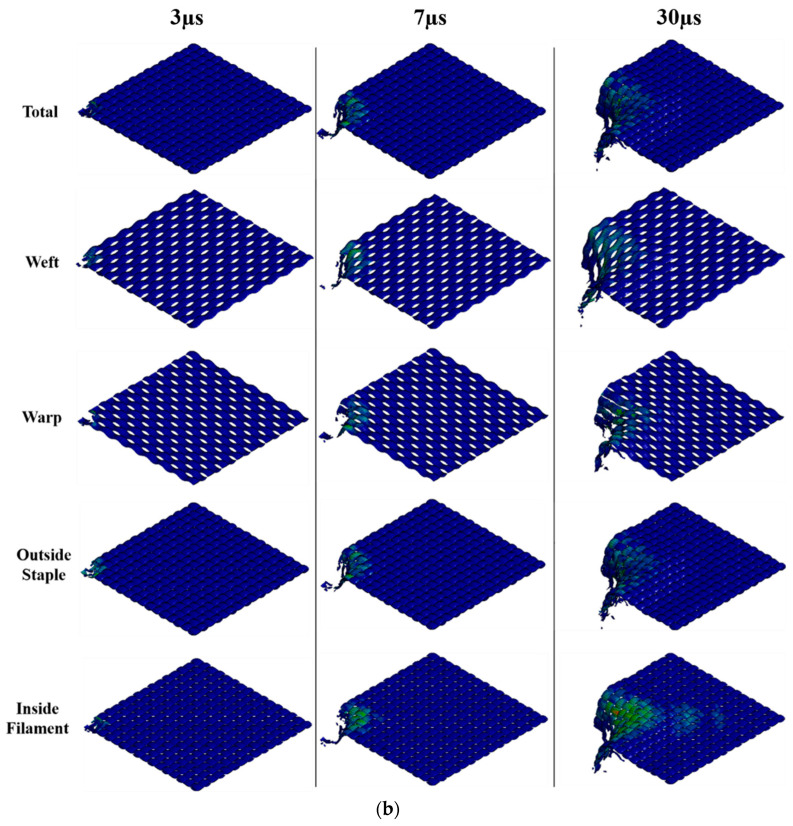

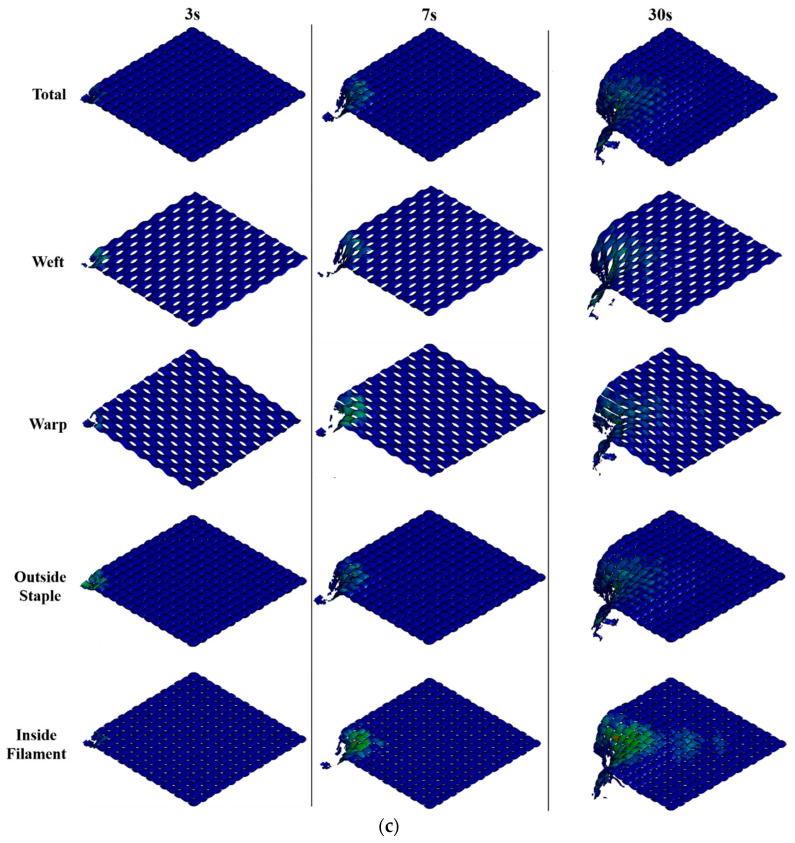
Ballistic penetration at three different time steps of 3 µs, 7 µs, and 30 µs: (**a**) F_a_, (**b**) F_b_, (**c**) F_c_.

The ballistic penetration of F_a_, F_b_, and F_c_ at these three typical times steps (3 μs, 7 μs, and 30 μs) is clearly illustrated in Figure 13.

In the first stage from the start point to 3 μs, the projectile hits the largest penetration resistance and is going to penetrate the whole structure. It can be noticed that stretching is the major formation in the structure to withstand the largest initial penetration in the beginning. All the related yarns are resisting this penetration in the formation of stretching, and some locations (especially the hit area) are further breaking. Though F_b_ and F_c_ are composed of CSC yarns with more complicated yarn structure, they demonstrate similar characteristics, as the outside staple and inside filament show similar responses to ballistic penetration.

In the second stage from 3 μs to 7 μs, the formation becomes quite varied. Cone deformation, yarn pull-out, and yarn fracture all become apparent and can be observed when the projectile further penetrates and tries to pass through F_a_, F_b_, or F_c_.

In the third stage, for all fabrics, the impact velocity is inclined to be stable, as the fabric has been penetrated completely and the penetration resistance tends to disappear.

The characteristic of acceleration is an important index to reflect the details of ballistic performance. As shown in Figure 12, it can be noticed that the largest increasement of acceleration appears for all fabrics (including F_a_, F_b_, and F_c_) during the first stage, namely, from the start point to around 3 μs. This is because in the beginning, the projectile has the largest energy to hit the fabric, and the yarn in the fabric receives the stress from the projectile and is inclined to offer penetration resistance.

In the second stage (from 3 μs to 7 μs), the acceleration for F_a_, F_b_, and F_c_ reduces apparently. This is because, during this time, the projectile has penetrated the fabric and passes through it. This may indicate that only the side surface of the projectile could have a little effect on a small area of the fabric which directly touches it; some phenomena such as broken or damaged yarns can be found in this area. The top of the projectile does not influence the fabric, though this part carries the most energy. Then comes the third stage; the acceleration reduces slightly and tends to zero.

The interesting point is that more time is needed for F_b_ than F_a_ and F_c_ to bounce back, i.e., around 7 μs for the front compared to 5 μs for the latter two. This tendency in the acceleration is due to the penetration resistance, which, again, proved the superiority of using staple fiber, especially aramid staple fiber, in the construction to improve the friction and therefore to enhance the interaction of the ballistic mechanism.

## 5. Conclusions

This paper aimed to enhance the ballistic performance of ballistic fabrics with the introduction of CSC yarns, and the results were quite positive. The energy absorption capability of the whole ballistic fabric could be apparently improved. Ballistic tests and FEA models were used to analyze the ballistic performance of these ballistic fabrics from the perspective of both experimental and theoretical views. The major contributions of this research are listed as follows:

(1) Three different ballistic fabrics were successfully manufactured, including two new types with the usage of CSC yarns. F_a_ was the conventional woven type with the usage of Kevlar^®^ 29 filament yarns. F_b_ was woven by CSC yarns with aramid staple fiber. F_c_ was also woven by CSC yarns with polyester staple fiber.
(2) These three types of ballistic fabrics, F_a_, F_b_, and F_c_, were ballistically tested. The experimental results showed that F_b_ and F_c_ have better ballistic performance than F_a_ as the energy absorbed by F_b_ and F_c_ is apparently higher than that absorbed by F_a_. This may indicate that the usage of CSC aramid yarns could, indeed, have a positive influence in improving the capability of energy absorption in each woven type. In addition, F_b_ is better than F_c_, which further indicates that aramid staple fiber is better than polyester staple fiber in the compound (CSC) yarn for providing better energy absorption capability. This may demonstrate that, except for the factor of friction, different composition of staple fiber could be another factor to influence the ballistic performance of the whole woven structure.
(3) The details of energy absorption for these three different ballistic fabrics were further investigated by FEA models. The theoretical results show a good coincidence with experimental results: regardless of which time duration, F_b_ is always higher than F_c_ and F_a_. The peak value of energy absorption in total for F_b_ is always stronger than F_b_ and F_c_. In addition, F_b_ is better than F_c_ in the energy absorption in total. It could be clearly noticed that the introduction of CSC yarns could highly enhance the capacity of energy absorption. FEA models also show the comparisons between V_0_ and energy after normalization calculated by FEA models for F_a_, F_b_, and F_c_. The results demonstrate that with the increase in V_0_, V_1_ increases correspondingly with no fluctuations, very similar to the trend illustrated from the experimental data. This may prove that the impact velocity before and after penetration is unlike to be influenced by a certain velocity, verified by both experimental and theoretical tests.
(4) The penetration resistance of these fabrics was also theoretically analyzed in detail. For the overall trend of velocity variation, F_a_, F_b_, and F_c_ show the same trends: decreasing sharply in the beginning, bouncing back later, and remaining stable finally. The relationship between the acceleration and the time history was also investigated. The theoretical analysis shows that F_b_ and F_c_ have better penetration resistance than F_a_, and F_b_ is the best due to the superiority of its structure and the usage of aramid staple fiber, which complies with the discovery in the research of energy absorption.

## Figures and Tables

**Table 1:** Specification of ballistic fabrics.

Fabrics	Composition of Yarn	Structure	Areal Density (Warp × Weft/cm^2^)	Weight (g/m^2^)
F_a_	Kevlar^®^ 29 filament yarn	Plain	14 × 14	135
F_b_	CSC yarn (core: Kevlar^®^ 29 filament yarn; spun with: aramid staple fiber)	Plain	14 × 14	140
F_c_	CSC yarn (core: Kevlar^®^ 29 filament yarn; spun with: polyester staple fiber)	Plain	14 × 14	145

**Table 2:** Results of ballistic tests.

Sample	Impact Velocity *V*_0_ (m/s)	Exit Velocity *V*_1_ (m/s)	Loss of Kinetic Energy Δ*E* (J)	Areal Density ρ (g/m^2^)	Normalization Δ*E*/ρ (Jcm^2^/g)
F_a_-1	246.6	246.5	19.7	135	146.1
F_a_-2	262.8	262.7	21		155.7
F_a_-3	290.8	290.7	23.3		172.3
F_a_-4	317.2	317.1	25.4		187.9
F_a_-5	324.6	324.5	26		192.3
F_b_-1	246.1	246	29.5	140	210.9
F_b_-2	260.2	260.1	29.1		208.1
F_b_-3	285.1	285	31.9		228
F_b_-4	300.6	300.5	33.7		240.4
F_b_-5	327.1	327	39.2		280.3
F_c_-1	231.3	231.2	25.9	145	178.6
F_c_-2	247	246.9	25.7		177.1
F_c_-3	275.3	275.2	28.6		197.4
F_c_-4	297.8	297.7	31		213.5
F_c_-5	327.7	327.6	36.7		253.1

Remarks: F_a_-1: No. 1 of single-layer F_a_ sample, F_b_-1: No. 1 of single-layer F_b_ sample, F_c_-1: No. 1 of single-layer F_c_ sample.

**Table 3:** Results calculated by FEA models.

Sample	Impact Velocity *V*_0_ (m/s)	Exit Velocity *V*_1_ (m/s)	Loss of Kinetic Energy Δ*E* (J)	Areal Density ρ (g/m^2^)	Normalization Δ*E*/ρ (Jcm^2^/g)
F_a_-1	246.6	246.53	13.8	135	102.3
F_a_-2	262.8	262.72	16.8		124.6
F_a_-3	290.8	290.71	20.9		155.1
F_a_-4	317.2	317.1	25.4		187.9
F_a_-5	324.6	324.5	26		192.3
F_b_-1	246.1	245.97	25.6	140	182.8
F_b_-2	260.2	260.08	25		178.4
F_b_-3	285.1	284.98	27.4		195.5
F_b_-4	300.6	300.48	28.9		206.1
F_b_-5	327.1	326.97	34		242.9
F_c_-1	231.3	231.18	22.2	145	153.1
F_c_-2	247	246.89	21.7		149.9
F_c_-3	275.3	275.19	24.2		167
F_c_-4	297.8	297.69	26.2		180.7
F_c_-5	327.7	327.58	31.5		216.9

Remarks: F_a_-1: No. 1 of single-layer F_a_ sample, F_b_-1: No. 1 of single-layer F_b_ sample, F_c_-1: No. 1 of single-layer F_c_ sample.

## Data Availability

All data generated or analyzed during this study are included in this published article.
